# Wonderfully Made

**Published:** 1861-04

**Authors:** 


**WONDERFULLY MADE.**—The microscope discovers to us that the mold on bread and other provisions in damp, warm weather is a dense forest in miniature, and has its regular trees and trunks and branches, with their buds and leaves and flowers and fruit. It proves that the butterfly is covered with feathers so beautiful and gorgeous in their tints, that no painter can ever hope to equal them; each hair is seen to be a hollow tube, and the softly-feeling skin is overlaid with scales like those of a fish; so tiny are they, that a single grain of sand will cover dozens of them, and each scale in turn covers hundreds of pores, to protect their mouths from being plugged up by dust and dirt, and to shield them from disorganization by overheat, or destructive chilliness by sudden blasts from the fierce cold of winter. The thinnest gauze of our stores, when thrown over the face, exposed to a keen and bitter wind, affords a degree of relief scarcely credible from so frail a material; but a scale of the skin is of the nature of horn, and alike impervious to dust, and wind, and water; and yet being firmly attached to the body at one edge only, the perspiration oozing out from under it, raises the free edge, and thus escapes from the body, loaded with its impurities and its wastes, to the average extent of two or three pounds a day. Laborers who do the blowing in glass-works lose by weight very near four pounds in a single hour! the perspiration streaming through twenty-five hundred pores to each square inch of the human body, or seven millions of pores in all, which, if joined together, would make a canal of twenty-eight miles in length. (See our book on SLEEP.) If a fish is deprived of its scales, it will be chilled to death; and reasoning analogically, and knowing too, that human skin-scales are dissolved by the alkali of the soap, a man may wash himself too much in soap and water, may actually wash away the scales of his body, leaving the pores so unprotected against heat and cold and obstructions, that death will inevitably ensue: indeed, physiological research proves, that if a third of the skin is removed from the body by scalding or otherwise, a fatal termination is unavoidable. Observant persons know how soon the skin becomes pale, shriveled, and tender, even on the harder hands, if kept a great deal in common cold water. These are suggestive considerations for those who have been led by plausible ignorance to believe that continual water sloshings are indispensable to health and longevity.

